# The effects of sex and load on quantifying the bilateral force deficit during an upper body Wingate test

**DOI:** 10.3389/fspor.2024.1446909

**Published:** 2025-01-03

**Authors:** Angie K. Antolinez, Philip F. Edwards, Michael W. R. Holmes, Duane C. Button

**Affiliations:** ^1^ School of Human Kinetics and Recreation, Memorial University of Newfoundland, St. John's, NL, Canada; ^2^ Faculty of Applied Health Sciences, Brock University, St. Catharines, ON, Canada; ^3^ Faculty of Medicine, Memorial University of Newfoundland, St. John's, NL, Canada

**Keywords:** bilateral deficit, cycling, fatigue, upper body, velocity

## Abstract

**Introduction:**

The bilateral deficit (BLD) is a reduction in the amount of force during a bilateral task vs. the total force from the unilateral limbs performing the same task. We quantified the BLD during an upper body Wingate Anaerobic Test (WAnT) and evaluated the influence of sex and load on the BLD in force.

**Methods:**

Eighteen participants performed maximum handgrip strength, voluntary isometric contractions (MVIC), and three 30s WAnTs. In each session they completed the tasks with the dominant-arm, non-dominant arm and with both arms, randomly. WAnT intensities were 3, 4, and 5% body weight (BW). Instantaneous force data was used to calculate the BLD.

**Results:**

Males showed greater (*p* < .001) BLD of force at 3, 4, and 5% BW than females by −17, −27.6 and −36%, respectively and had a greater (*p* < .001) BLD of force than females throughout time points 1–10 s, 11–20 s, and 21–30 s by −16, −29 and −35%, respectively. Females showed a difference (*p* < .001) in BLD of force between loads (−19% at 3%, −10% at 4% and +7% at 5%). Males had an increase (*p* < .001) in BLD of force from the beginning to the end of the WAnT starting with −18% (1–10 s), −38% (11–20 s) and −40% (21–30 s). WAnT had the highest BLD, followed by MVIC and grip strength.

**Discussion:**

BLD in force is present during WAnTs and the sex-load interaction is important for determining this BLD during this maximal cycling test. Thus, when developing training or rehabilitation programs related to BLD in force, sex, load and exercise type should be taken into consideration.

## Introduction

The bilateral deficit (BLD) is a physiological phenomenon characterized by a reduction in performance during a bilateral motor task compared to individual unilateral performance combined during the same motor task (1). The BLD phenomenon is complex, highly variable, and subject to training adaptations. It has been shown to exist in many different motor outputs (1–4). Numerous studies have explored the BLD during isometric contractions (5–12) and dynamic contractions (2, 13–15). The average magnitude of the BLD during dynamic and isometric contractions is approximately 11.7% and 8.6%, respectively (1). However, there are large variations in the magnitude across studies and motor outputs. In some studies participants have shown bilateral facilitation (BLF), a physiological phenomenon characterized by an increase in performance during a bilateral motor task compared to individual unilateral performance combined during the same motor task, which is the opposite of BLD (1, 16, 17). The mechanisms behind BLF remain unclear, however, the effect of fatigue during unilateral force and power production tasks might be one reason why during bilateral fatiguing tasks there is facilitation of force production from one arm to the other (18).

One study that explored the BLD during a cyclical movement (19), found a BLD in peak power and total mechanical work during leg cycling Wingate Anaerobic Tests (WAnT), but BLD in force was not measured. To our knowledge, no studies have determined if there is a BLD in force during a cyclical movement, and no studies have assessed the BLD phenomenon using an upper body cyclical movement. It has been stated that the BLD appears to be limited to twin-synchronous movements but not simultaneous flexion and extension, which can be seen during asynchronous cycling movements (1, 20); however, there is little evidence to support this claim.

Sex is another factor that influences the BLD phenomenon. Mechanical, physiological and psychological factors that influence the force and power production in males vs. females have been explored for different motor outputs (21). Regarding sex differences, few studies have compared the BLD between males and females. To our knowledge, the first study exploring the BLD between sexes was done by Ye et al. (17) who compared maximal voluntary isometric contractions (MVIC) of the elbow flexors and the finger abductors. Both males and females showed a BLD in the elbow flexor force, whereas only males showed a BLD in finger flexor force. Interestingly, women show a non-significant BLF in finger flexor force. Carr et al. (22) explored the BLD in maximal voluntary contractions but showed no sex differences. However, absolute values of BLD were not reported. The most recent study (16) accounting for sex differences compared the BLD during a countermovement jump between male and female athletes and its correlation with change of direction performance. They found that males had a BLD while females showed BLF and that BLD correlated with a change of direction only in males. No previous study has explored sex differences during a cyclical power production task. However, most studies do suggest that males have a greater BLD than females.

The BLD implications has been mainly explored in athletic performance. Železnik et al. (23), discussed that BLD, as calculated from vertical jump outcomes, was found to be positively correlated with the ability to change direction quickly in volleyball, basketball, tennis, and among students, but not in soccer. However, there is little evidence of sport specific implications of BLD, as well as a scarcity of literature on the implication of BLD in injury and recovery. This study aimed to quantify the BLD in force during an upper body WAnT and determine if the magnitude of the BLD, if one existed, was affected by sex and load (3, 4, and 5% of the participant's body weight). Because there are no established relationships between power production tasks and fatigue in terms of the magnitude of the BLD of force, we also compared the change in BLD of force across time during the WAnT (1 s–10 s vs. 11 s–20 s vs. 21 s–3 0 s). Incorporating upper body WAnTs at different intensities and measuring the BLD of force at three different time periods throughout the WAnT will help determine the effects of upper limb fatigue on the BLD of force. Finally, the effect of task specificity (cycling vs. isometric contractions) and the position during arm cycling (12 o'clock position vs. 6 o'clock position) on the BLD of force were also determined. These positions have been shown to have the highest muscular and neural activation during arm cycling for the main agonist and antagonist muscles during elbow flexion and extension (24). Measuring forces at two different positions, 12 o'clock, and 6 o'clock, where the triceps brachii and biceps brachii muscles are contributing the most to the movement (25), respectively, will help to determine if there are intermuscular differences in the magnitude for the BLD of force. It was hypothesized that (1) there will be a significant BLD in force during arm cycling and (2) the magnitude of the BLD in force will be affected by sex, intensity (% of body weight), the development of fatigue during the WAnT, and position. Portions of the data, including power, fatigue indices, force outputs and biomechanics, that were collected during the current research study, have been previously published (26).

## Methods

### Ethical approval

Before data collection, participants were informed of all potential risks and benefits of the study via verbal and written explanation and were given an opportunity to ask questions. All participants then gave written informed consent. This study was approved by the Interdisciplinary Committee on Ethics in Human Research at Memorial University of Newfoundland (ICEHR No. 20230904-HK).

### Participants

Eighteen (9 males and 9 females) healthy adults volunteered for the study (Table 1). Experimental methods and participant data for power output, fatigue indices, force output and biomechanics have already been published elsewhere (26). Based on Antolinez et al. (26), the calculation from G power (27) using F tests, specifically, a two tailed repeated measures ANOVA with an alpha of 0.05 and a power 0.8, recommended a minimum sample size of 16 participants. Participants had no prior experience with upper body WAnTs. Participants with upper limb injury in the last six months or pain that prevented them from completing vigorous exercise were excluded. Participants completed a Physical Activity Readiness Questionnaire (PAR-Q+) to ensure they could safely perform physical activity (28). Hand dominance was then determined using the Edinburgh Handedness Inventory (29).

**Table 1:** Demographic information of participants.

Sex	Age (years)	Height (cm)	Mass (kg)	BMI (kg/m^2)^	Handedness (R:right, L:left)	Net load (kg)	
Males (*n* = 9)	25.7 ± 5.2	176 ± 8	76.9 ± 34	24.7 ± 8.2	R = 8 L = 1	3% 4% 5%	2.3 ± 1 3.1 ± 1.4 3.8 ± 1.7
Females (*n* = 9)	25.8 ± 4.2	163.2 ± 13	66.8 ± 22	25.1 ± 3.7	R = 7 L = 2	3% 4% 5%	2 ± 0.6 2.7 ± 0.9 3.3 ± 1.1

### Experimental set-up

#### Arm cycle ergometer

**Figure 1:**
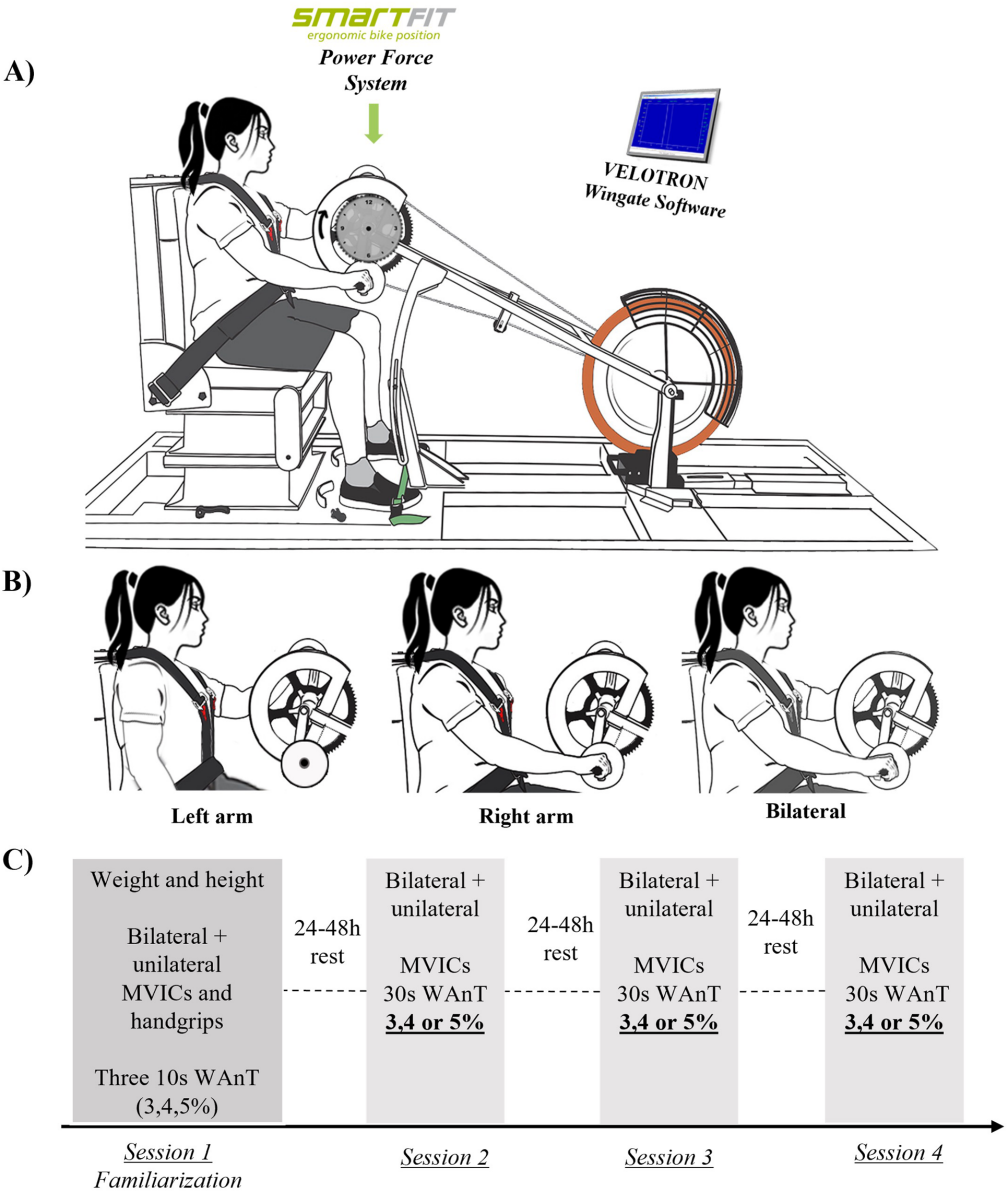
Experimental set-up and protocol. **(A)** Represents the upper body bilateral WAnT set-up. Wingates and MVICs were recorded in this position bilaterally. The graph shows the smart fit power force recording system, the modified Velotron upper body cycle-ergometer set-up and the software to perform the WAnT. **(B)** Unilateral WAnTs and MVICs were performed for each arm and the handgrip strength were performed in the same position holding a hand dynamometer bilaterally and/or unilaterally. **(C)** Experimental protocol.

All arm cycling trials were performed on a Velotron cycle ergometer (Dynafit Pro, RacerMate, Seattle, Wash., USA) modified for arm cycling Figure 1. Participants were seated in a padded armless chair with their upper body strapped to the chair and their feet strapped to the floor. The height of the ergometer was adjusted so the center of the crankshaft was approximately in line horizontally with the participant's acromion. The padded chair distance was manipulated for each participant and positioned to ensure no reaching for the arm cranks at full elbow extension. The ergometer height and chair distance were recorded for each participant, and these values were used in all sessions. The hand cranks were locked 180° out-of-phase to perform asynchronous cycling.

#### Force data

Instantaneous force data (N) on the pedal axis was recorded using Powerforce Smartfit sensors (Radlabor GmbH, Freiburg, Germany). All signals were sampled at 500 Hz analog-digital converted and recorded using IMAGO® software (IMAGO Technologies GmbH, Freiburg, Germany). The propulsion force or effective force tangential to the crank's movement direction was recorded during all WAnTs.

#### Maximal grip strength

Maximal grip strength (kg) was collected bilaterally and unilaterally with a Smedley Digital Hand Dynamometer (Model: 12-0286, Baseline® Digital hand dynamometer, White Plains, New York, USA) in two static asynchronous cycling positions. The first-hand grip test was recorded bilaterally, with the dominant arm at 12 o'clock and the non-dominant arm at 6 o'clock. Two trials were performed. The second-hand grip test was recorded bilaterally in the opposite position; two trials were performed. Unilateral hand grips were also performed at the 12 and 6 o'clock positions with both arms. A total of 12 measurements of maximal grip strength were performed. Participants held the contraction for five seconds each while verbal encouragement was given, with two trials on each position and a 60-s rest between trials.

#### Maximal voluntary isometric contractions (MVIC)

All MVICs were performed bilaterally and unilaterally on the cycle ergometer with the wheel locked in place. MVICs were performed in the cycling position at 12 o'clock and 6 o'clock. At 12 o'clock (elbow extension: pushing), the shoulder and elbow were flexed approximately 90° with some shoulder abduction. At 6 o'clock (elbow flexion: pulling), the shoulder was in a neutral position, and the elbow was flexed approximately 90°. At 12 and 6 o'clock the wrist was 90° pronated. The BLD in force was named based on the position of the dominant arm; for example, the pushing MVIC corresponds to the dominant arm at 12 o'clock while the non-dominant arm is at 6 o'clock. At 12 o'clock and 6 o'clock, the wrist was pronated and holding the crank. The participants performed 3 MVICs in each position, with a 60-second rest between contractions, and were held for five seconds each while verbal encouragement was given (26). The force data (N) was recorded with the Smart Fit Power system.

### Arm cycling WAnT

The arm cycling WAnT protocol consisted of a brief 10-second warm-up at a 60-rpm cadence, followed by a visual and verbal three-second countdown prior to the electro-mechanical brake applying 3, 4, or 5% of the participant's BW. The resistances 3, 4, and 5% of participant BW for the WAnTs were chosen based on previously published data (26). Once the resistance was applied the participant cycled at maximal effort for 30 s. During unilateral cycling WAnTs, participants were instructed to rest their inactive contralateral arm on their lap to minimize any potential effects of movement of the contralateral arm on the unilateral arm cycling WAnT. Visual cadence feedback was provided on a computer screen in front of the participants. Verbal encouragement was provided throughout the 30 s test. Crank force and performance data were recorded during the 30 s test.

### Experimental protocol

#### Familiarization session

During the familiarization session, maximal grip strength tests were performed, weight and height were measured to set up the WAnT load percentages, and then participants performed six 5-s arm cycling sprints at 3% BW, two sprints each with the dominant, non-dominant, and both arms, in a randomized order. After completing the familiarization session, participants were given 48 h of rest before their first experimental session.

#### Experimental sessions

Participants performed three 30-second upper body WAnTs during each of the three experimental sessions. MVIC (N) was recorded at the beginning of each experimental session. One WAnT was performed with both arms, the dominant arm and the non-dominant arm, in random order. See Figure 1 for the experimental set-up. Twenty-minute rest intervals were provided between each WAnT to minimize the effects of fatigue from the prior WAnT. The intensity of the WAnTs was randomly determined before the session as either 3% BW, 4% BW, or 5% BW. The resistances of 3, 4, and 5% BW for the WAnTs were chosen based on previously published data (26).

### Data analysis

All data analysis was performed offline using MATLAB (Version R2022b, The Mathworks, Natick, MA, USA). All force data were filtered using a 4th-order lowpass Butterworth filter with a cut-off frequency of 50 Hz. Force data (N) were resampled to record instantaneous force from each part of the cycle (0° to 360° or 12 o'clock position to 12 o'clock position) for each individual cycle during the WAnT. A digital trigger on the force sensor indicated when the right-hand crank was at the 6 o'clock position by displaying a “1” value. A “0” value indicated every other position. Each cycle during the WAnT was quantified by finding a 1 value and then finding a subsequent 1 value. This constituted a full cycle. The instantaneous position of the crank was determined by calculating the total number of samples in the cycle and determining what percentage of the full cycle the crank was currently positioned.

The 12 o'clock (0°) and the 6 o'clock positions (180°) were used for data analysis because these two positions represented the two peaks of force production during the upper body WAnTs. The peak propulsive force at the 12 o'clock and 6 o'clock positions were determined for all cycles, at all intensities from 1 s to 10 s (10 s), 11 s to 20 s (20 s), and 21 s to 30 s (30 s) for every WAnT. The average of the peak forces at these positions was then used to calculate the bilateral deficit. The bilateral deficit was calculated as follows:

(1) $$\mathrm{BI}(\text{\%})=\left(100\times \frac{\mathrm{Sum}\;\mathrm{of}\;\mathrm{the}\;\mathrm{average}\;\mathrm{peak}\;\mathrm{bilateral}\;\mathrm{forces}}{\mathrm{Sum}\;\mathrm{of}\;\mathrm{the}\;\mathrm{average}\;\mathrm{peak}\;\mathrm{unilateral}\;\mathrm{forces}}\right)-100$$

For example, to calculate the bilateral deficit in force at the 6 o'clock position, the sum of the average peak bilateral forces at the 6 o'clock position for the dominant and non-dominant arms and the sum of the average peak unilateral forces at the 6 o'clock position for the dominant and the non-dominant arms were determined, and the bilateral index (BI) was calculated using Equation 1. The same formula was used to calculate the bilateral deficit for maximal grip strength and the MVICs. A negative percentage indicates a BLD and a positive percentage indicates a BLF of force.

### Statistical analysis

**Figure 2:**
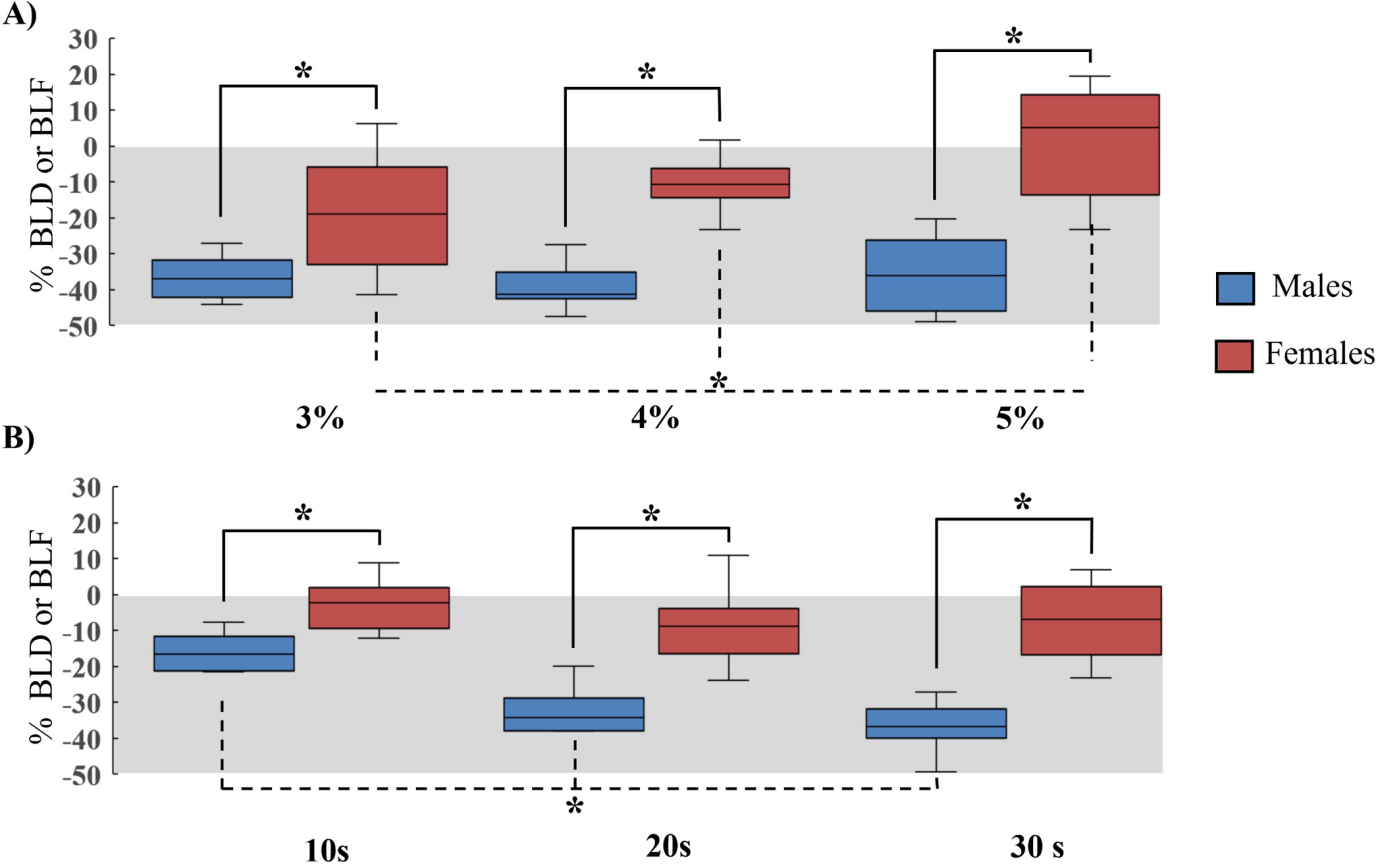
Sex-load differences of the BLD during an upper body WAnT. The *Y* axis represents the BLD/BLF as a %. Males are represented in blue and females in red. **(A)** The left, middle and right graphs represent the 3,4 and 5% WAnT loads, respectively. **(B)** The left, middle and right graphs represent the average BLD during the 1-10s, 11-20s and 21-30s time points, respectively. The grey shadowed area indicates the area of bilateral deficit (BLD < 0). Significant differences between sexes are indicated with a solid line. Significant differences between loads and time points are indicated with the dashed line. *(*p* < .05).

**Figure 3:**
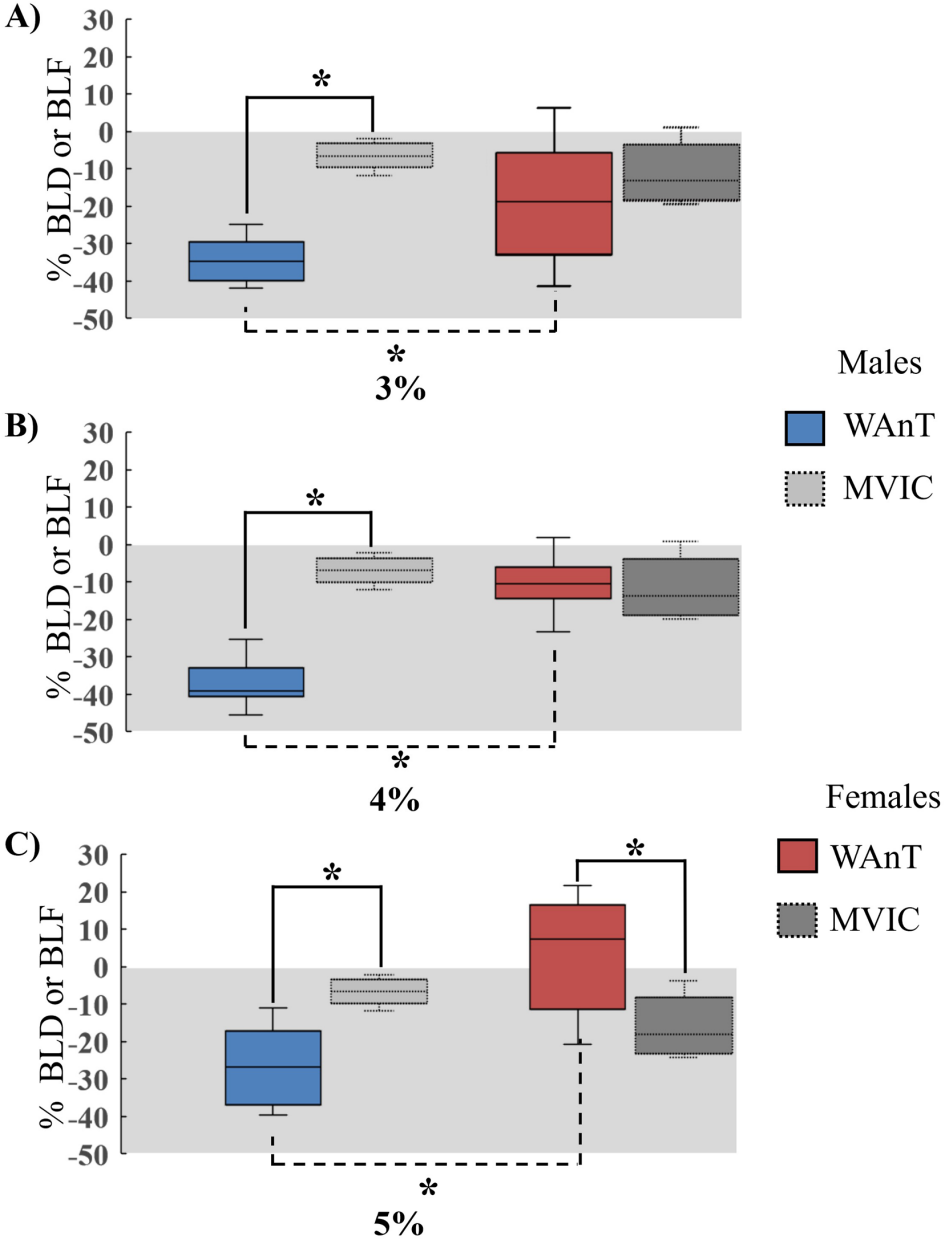
Bilateral deficit during an upper body WAnT vs MVIC in males and females. **(A)** 3% load **(B)** 4% load **(C)** 5% load. BLD during the WAnTs are represented by the blue and red boxes for males and females, respectively and the BLD during MVIC with the dotted line and represented by the light grey and dark grey boxes for males and females, respectively. The grey shadowed area indicates the area of bilateral deficit (BLD < 0). Significant differences between tasks are indicated with a solid line. Significant differences between sexes are indicated with the dashed line. *(*p* < .05).

Statistical analyses were completed using SPSS 28.0 (SPSS for Windows, IBM Corporation, Armonk, New York, USA). The normality of the data was assessed using both Shapiro–Wilk, and Kolmogorov–Smirnov tests, and it was found that all the variables were normally distributed (*p* > 0.05). Individual repeated measures ANOVAs were performed for each condition: WAnTs, MVIC, and grip strength. For the WAnTs, the model was LOAD (3, 4, and 5% BW) × TIME (10 s, 20 s, 30 s) × POSITION (12, 6 o'clock). For the MVIC and grip strength, the model only included POSITION. Three two-way ANOVAs (one for each load), including CONDITION × POSITION, were also performed to compare the differences between conditions. For all ANOVAs, SEX (males vs. females) was determined as the between-subjects factor. If violating the assumption of sphericity, *p* values were adjusted using the Greenhouse–Geisser correction. Statistical significance for main tests was set at *p* ≤ 0.05. In the event of a statistically significant ANOVA outcome, pairwise comparisons were completed *post hoc* using the Bonferroni correction. The text and tables show data as mean ± SD. Partial eta-squared (*η_p_*^2^) measures indicating the magnitude of changes associated with significant main effects were provided and reported as small (<0.01), medium (≥0.06), or large (≥0.14) (30). Figures 2 and 3 are represented as box and whisker plots.

## Results

Demographic information about the participants is found in Table 1. For absolute values of the BLD of force during upper body WAnTs between loads and times, force during grip strength and MVICs see Table 2. The force values (N) of the dominant vs. non-dominant arm during the unilateral and bilateral upper body WAnT are reported in Table 3. Unilateral and bilateral forces of grip strength (kg) and MVIC (N) are reported in Table 4. Hereafter, all values representing BLD and BLF will be reported as negative and positive, respectively.

**Table 2:** **(A)** Bilateral index during a wAnT at 3,4 and 5% body weight for males and females during the first 10 s (1–10 s), the second 10 (11–20 s) and the last 10 s (21–30 s) of the test. **(B)** Bilateral index at 12 and 6o’clock positions for hand grips and MVIC.

(**A**)												
T	3%			4%			5%			Differences between loads (*P*-values)		
	Males	Females	*P*-values	Males	Females	*P*-values	Males	Females	*P*-values	%	Males	Females
10 s	−19.7 ± 8.4	−8.7 ± 13.4	.031*	−18.9 ± 9.0	−6.3 ± 13.2	***0.10***	−17.6 ± 7.9	6.8 ± 19.7	.002*	3–4	***1***.***00***	***1***.***00***
										4–5	***1***.***00***	.***125***
										3–5	***1***.***00***	.***020#***
20 s	−37.99 ± 12.8	−18.85 ± 21.1	.017*	−42.4 ± 8.9	−18.9 ± 10.7	<.001*	−34.5 ± 11.1	10.8 ± 22.3	<.001*	3–4	.***83***	***1***.***00***
										4–5	.***38***	***<.001#***
										3–5	***1***.***00***	***<.001#***
30 s	−51.4 ± 10.3	−29.7 ± 20.6	.005*	−52.3 ± 8.6	−5.5 ± 22.4	<.001*	−34.9 ± 21.8	3.9 ± 20.5	<.001*	3–4	***1***.***00***	***<.001#***
										4–5	.***033#***	.***408***
										3–5	.***229***	.***004#***
(**B**)												

Position	Handgrips			MVIC		
	Males	Females	*P*-values	Males	Females	*P*-values
12 o’clock	−1.5 ± 3.2	−3.5 ± 7.04	.***43***	−8.1 ± 4.3	−8.6 ± 7.8	***0***.***86***
6 o’clock	−1.7 ± 3.9	−.6 ± 8.6	.***74***	−9.6 ± 4.6	−14.3 ± 8.6	.***17***

*P*-values (in bold and italicized) represented with an * indicate significant sex differences on each load at each period of time and *P*-values represented with # indicate load differences within the respective sex and time point. Time (T).

**Table 3:** Average forces (N) produced during a wAnT by each sex at each load (3,4,5% of body weight) during 1–10 s,11–20 s and 21–30 s. First column represents the forces during unilateral WAnTs of the dominant arm: D and the non-dominant arm: ND. The second column shows the total force when adding the unilateral WAnTs and the third column is the total force (D + ND) during a bilateral WAnT.

Load	Time point	Unilateral wingate		Unilateral wingate D + ND		Bilateral wingate D + ND	
		Males	Females	Males	Females	Males	Females
3%	10 s	D: 129.2 ± 33.9 ND: 137.5 ± 42.4	D: 60.9 ± 36.3 ND: 60.3 ± 28.3	266.8 ± 38.2	121.2 ± 32.3	218.1 ± 26.8	109.5 ± 28.4
	20 s	D: 131.1 ± 43.2 ND: 152.8 ± 44.1	D: 72.6 ± 30.2 ND: 74.8 ± 35.7	283.8 ± 43.6	147.4 ± 33	171.1 ± 18.8	114.9 ± 25
	30 s	D: 133.5 ± 48.2 ND: 171.1 ± 52.8	D: 82.1 ± 34.3 ND: 89.1 ± 45	304.6 ± 50.5	171.1 ± 39.6	143.2 ± 20.1	119.3 ± 27.5
4%	10 s	D: 141.4 ± 32.5 ND: 152.5 ± 45.3	D: 61.3 ± 31.1 ND: 55.3 ± 22.4	294.1 ± 38.9	116.7 ± 26.8	236.5 ± 26.3	108.5 ± 25.9
	20 s	D: 173.6 ± 48.9 ND:203.2 ± 69.7	D: 84.6 ± 31.1 ND: 73.3 ± 28.3	376.9 ± 59.3	157.9 ± 29.6	210.1 ± 20.9	135.6 ± 32.1
	30 s	D: 197.2 ± 45.5 ND: 222.7 ± 66.2	D: 99.6 ± 30.4 ND: 76.3 ± 31.9	419.9 ± 55.9	176 ± 31.2	195.2 ± 27.7	162.7 ± 31.8
5%	10 s	D: 154.3 ± 49.8 ND: 160.9 ± 54.2	D: 60.7 ± 35.3 ND: 55.3 ± 35.2	315.2 ± 52	116.1 ± 35.3	263.7 ± 37.4	116.2 ± 28
	20 s	D: 204.3 ± 65.1 ND: 214.1 ± 69.2	D: 77.9 ± 32.3 ND: 72.8 ± 45.9	418.4 ± 67.2	150.7 ± 39.1	272.5 ± 42.6	165.8 ± 42.2
	30 s	D: 224.8 ± 59.1 ND: 221.3 ± 74.1	D: 92.6 ± 38.6 ND: 82.1 ± 39.9	446.1 ± 66.6	174.8 ± 39.3	282.5 ± 50.5	172.1 ± 36.2

**Table 4:** Forces produced by each sex during **(A)** hand grips (kg) and **(B)** MVIC (N) at each position. First column represents the unilateral forces of the dominant arm: D and the non-dominant arm: ND. The second column shows the total force when adding the unilateral forces and the third column is the total force (D + ND) during a bilateral MVIC or handgrip.

(**A**)						
Position	Unilateral handgrip		Unilateral handgrip D + ND		Bilateral handgrip D + ND	
	Males	Females	Males	Females	Males	Females
12 o’clock	D: 36.6 ± 5.4 ND: 34.5 ± 6.4	D: 23.5 ± 4.6 ND: 21.1 ± 3	74.2 ± 9.07	45.4 ± 6.27	72.6 ± 9.7	43.9 ± 7.6
6 o’clock	D: 37.7 ± 4.3 ND: 37.6 ± 5	D: 24.0 ± 4.5 ND: 21.9 ± 2.6	72.2 ± 10	45.7 ± 6.8	71.6 ± 9.1	44.7 ± 7.4
(**B**)						

Position	Unilateral MVIC		Unilateral MVIC D + ND		Bilateral MVIC D + ND	
	Males	Females	Males	Females	Males	Females
12 o’clock	D: 386.1 ± 24.4 ND: 397.5 ± 111.6	D: 190.6 ± 95.2 ND:183.1 ± 70.3	717.9 ± 168.6	305.8 ± 160.6	662 ± 170.7	275.1 ± 136.6
6 o’clock	D: 310.9 ± 54.5 ND:331.7 ± 72.6	D:154.6 ± 37.7 ND:153.4 ± 36.9	717.1 ± 148.2	318.5 ± 174.3	633.4 ± 170.7	267.9 ± 129.5

### BLD in an upper body WAnT

There were significant main effects for LOAD [*F*_(1.37, 22.05)_ = 19.28, *p* < .001 *η_p_*^2^ = .54], TIME [*F*_(2, 32)_ = 41.29, *p* < .001 *η_p_* = .72], and SEX [*F*_(1, 16)_= 49.5, *p* < .001 *η_p_*^2^ = .75] on BLD of force. *post hoc* comparisons showed that the BLD of force was −16.8% more at 3% than 5% BW (*p* < .001) and −13.1% more at 4% than 5% BW (*p* < .001). In terms of time, there was −17.5% more BLD of force in 30s (*p* < .001) and −12.9% more at the 20s than at the 10s period (*p* < .001). Males showed a −27% greater BLD of force than females (*p* < .001). There was no significant main effect of POSITION [*F*_(1, 16)_ = 1.99, *p* = .17 *η_p_*^2^ = .11] on BLD of force.

There were significant interactions for LOAD × SEX [*F*_(2 32)_= 5.54, *p* = .009 *η*_p_^2^ = .25], TIME × SEX [*F*_(2, 32)_= 12.63, *p* < .001 *η_p_*^2^ = .44], LOAD × TIME [*F*_(4, 64)_= 4.7, *p* *=* *.002 η_p_*^2^ = .22] and LOAD × TIME × SEX [*F*_(4, 64)_= 3.37, *p* = .014 *η_p_*^2^ = .17] on the BLD of force. Males showed more BLD of force at all loads than females [−17% more at 3% (*p* < .001), −27.6% at 4% (*p* < .001), and −36% at 5% (*p* < .001)]. Males also had a greater BLD of force than females throughout the time points of the WAnT [−16% more from 1 to 10 s (*p* < .001), −29% more from 11 to 20 (*p* < .001), and −35% more from 21 to 30 (*p* < .001)]. Males did not show a significant difference in BLD of force between loads (*p* = 1, *p* = .5, *p* = .11), but females did [−19% at 3% (*p* < .001), −10% at 4% (*p* < .001) and +7% at 5% (*p* < .001)]. Males had a significant increase in BLD of force from the beginning to the end of the test, starting with −18% (1–10 s), −38% (11–20 s) and −40% (21–30 s) (all *p* < .001). However, females did not significantly differ over time (*p* = 1, *p* = .12*, p* = .10). See Figure 2 for sex differences between loads and time points for BLD during the WAnT.

### BLD in MVIC

There was a significant main effect of POSITION [*F*_(1, 16)_ = 6.76, *p* *=* .019 *η_p_*^2^ = .29] on BLD of MVIC force. *post hoc* comparisons showed that BLD of force was −3.5% greater when pulling vs. pushing (*p* = .019). No significant effect was found for SEX [*F*_(1, 16)_ = .84, *p* = .37 *η_p_*^2^ = .05].

### BLD in grip strength

There was no significant main effects of POSITION [*F*_(1, 16)_ = .73, *p* *=* .40 *η_p_*^2^ = .44] or SEX [*F*_(1, 16)_ = .046, *p* = .83 *η*_p_^2^ = .003] on BLD of grip strength.

### Comparison of BLD between WAnT load and MVIC and grip strength

#### 3% load

There was a significant main effect for CONDITION [*F*_(2, 32)_ = 50.20, *p* < .001 *η_p_*^2^ = .75] on BLD. Pairwise comparisons showed that WAnT had the highest BLD (−27.7%) followed by MVIC (−10.1%) and grip strength (−1.8%) (*p* < .001). No significant effects were found for SEX [*F*_(1, 16)_ = 4.22, *p* = .057 *η_p_* = .20] or POSITION [*F*_(1, 16)_= 1.48, *p* = .24 *η_p_*^2^ = .08] for MVIC.

#### 4% load

There were significant main effects for CONDITION [*F*_(2, 32)_ = 69.38, *p* < .001 *η_p_*^2^ = .81] and SEX [*F*_(1, 16)_ = 24.82, *p* < .001 *η_p_* = .60] on BLD. There was also a significant interaction for CONDITION × SEX [*F*_(2 32)_ = 39.16, *p* < .001 *η_p_*^2^ = .71] on BLD. Pairwise comparisons showed that males had −27% greater (*p* < .001) BLD during WAnTs than females, with no significant sex differences in MVIC or grip. Males showed a significant difference between all conditions, with a −37% BLD during WAnTs, −8.8% during MVIC, and −1.5% for grip strength (*p* < .001). However, females only showed a significant difference between WAnTs (−10.3%) and grip strength (−2%) (*p* = .03) and between MVIC (−11%) and grip strength (*p* = .01). No significant effect was found for POSITION [*F*_(1, 16)_ = .54, *p* = .47 *η_p_*^2^ = .03].

#### 5% load

There were significant main effects for CONDITION [*F*_(2, 32)_ = 5.46, *p* = .009 *η_p_*^2^ = .25] and SEX [*F*_(1, 16)_ = 24.43, *p* < .001 *η_p_* = .60] on BLD. There was also a significant interaction for CONDITION × SEX [*F*_(2 32)_ = 25.85, *p* < .001 *η_p_*^2^ = .61] on BLD. Pairwise comparisons showed that males had a −36% greater BLD during WAnTs than females, with females showing +7.2% BLF (*p* < .001). There were no significant sex differences in the MVIC or grip strength. Males showed a significant difference between WAnTs (−29%) and MVIC (−8.8%) and grip strength (−1.5%) (*p* = .002) with no significant difference between MVIC and grip strength. Females showed a significant difference between WAnTs (+7.2%) and MVIC (−11.4%) (*p* = .003) and MVIC and grip strength −2.1% (*p* = .014). No significant effect was found for POSITION [*F*_(1, 16)_ = 1.28, *p* = .27 *η*_p_^2^ = .07]. Figure 3 represents the difference in BLD for males and females between WAnT and MVIC at each load.

## Discussion

In our study we showed that there was a significant BLD in force during arm cycling and that its magnitude was affected by sex, intensity (% of body weight), the development of fatigue during the WAnT, but not by the position during the cycle. No significant differences were found during the 12 o'clock vs. the 6 o'clock position, probably because during the task both arms muscle activation patterns are out of phase, since the cycling was asynchronous. Additional electromyography (EMG) studies are recommended to explore the activation patterns of the muscles and their relationship with BLD. To our knowledge, this is the first study to quantify the bilateral deficit phenomenon during an upper body WAnT (a maximal intensity rhythmic movement), considering factors such as sex, load, position, and time during the WAnT. The BLD of force was also compared between WAnT, asymmetric MVIC, and maximal grip strength. We found that BLD of force was load, sex, time and task dependent. Interestingly, the BLD for males remained at a similar deficit for all WAnT loads and at a higher deficit than females, whereas in females as WAnT load increased, they went from a BLD to a BLF. Furthermore, the BLD in men increased with time (i.e., the development of fatigue) at each load, but not in women. When comparing the WAnT vs. isometric conditions, the BLD of force was highest during the WAnT, followed by MVIC and grip strength. At all WAnT loads the BLD during the WAnT was greater than the isometric MVIC for males, however, for females there were no task-specific differences in BLD until completing a WAnT at 5% load. This work demonstrates that BLD during a WAnT is sex and load-dependent and differs greatly than the BLD during isometric tasks. This data clearly indicated that sex is a major factor in the BLD during maximal intensity arm-cycling.

### Task specific effects on BLD of force

Since the literature has not been consistent in terms of task specificity on the BLD of force, we compared cycling to an asynchronous isometric task and grip strength. Overall, we found that grip strength and MVIC ranged similarly to previous studies, between −1% and −15%; however, the BLD of force during WAnT ranged from −17% to as high as −50% towards the end of the test. The presence of a BLD in the upper body WAnT had not been explored previously. Initial studies on BLD of force were restricted to maximal contractions of twin synchronous movements, like simultaneous flexion or extension of homonymous limbs (10, 19). The most recent review on BLD showed that the most common tasks used to explore BLD of force are concentric/isokinetic contractions of the knee extensors, isometric grip strength and ballistic contractions in the lower limbs like squat jump or countermovement jump (1). However, the literature on exploring BLD of force during cycling tasks is scarce. The fact that we found a BLD of force of more than −20% in a task like arm cycling suggests a task-dependent factor in BLD, as suggested by Bishop et al. (31), who found differences in BLD in power and jump height during different types of jumps.

Studies on BLD of force of the upper body showed that it was present during different isometric contractions like shoulder flexion, elbow flexion, thumb adduction, finger abduction, and grip strength (32–34). The BLD in the upper body ranges from −1 to −26%, and specifically when performing grip strength, this variability has been attributed to postural differences between supine, seated, and standing or between supinated vs. neutral wrist position when recording grip strength (7, 14, 35). During dynamic concentric contractions of the upper body, it has been found that the BLD is −5.8% vs. −13.2% in the lower body (2, 36, 37). However, the task of the WAnT is based on performing at maximal force and at maximal velocity. The velocity of the contraction has been suggested to influence the BLD. Vandervoot et al. (38) compared 10 velocities during isokinetic hip and knee extensions and observed a linear increase in BLD of power as the contraction velocity increased. In the current study, we also showed a high level of BLD in force during high velocity movements of the arms.

### Influence of fatigue on the BLD of force during the wAnT

Another important factor to consider when measuring the BLD of force during the WAnT was time. The WAnT induces fatigue; thus, it was important to determine how the BLD of force changed over time or as fatigue progressed. We found that fatigue increased the BLD. However, this was only true for males; females did not show a significant difference in BLD as the test progressed and fatigue developed. However, previous research has been controversial in terms of the effect of fatigue on BLD. Vandervoort et al. (38) found that the BLD of force decreased with fatigue during repetitive leg extensions. Owings & Grabiner (15) contradicted the initial findings and found that fatigue increased the BLD of force during isokinetic knee extensions. Most recently, another study using repetitive leg extensions showed a decrease in BLD of force with fatigue (39). The current study demonstrated that during maximal arm-cycling sprinting the BLD increases with fatigue in men. Based on the aforementioned, it appears that the influence of fatigue on the BLD of force may be task-specific.

Fatigue can cause muscle inhibition and asymmetrical fatigue between limbs, further exacerbating BLD (40). The effect of fatigue on BLD is more pronounced during high-load tasks, where the central nervous system's ability to effectively coordinate bilateral movements is further compromised (41). However, well-trained individuals tend to experience smaller BLDs even under fatigue, due to enhanced motor unit recruitment and coordination (42). Overall, fatigue can amplify BLD by reducing efficiency in neural control and muscle function, especially under high-exertion conditions (43).

### Sex differences in BLD of force

We found sex-related differences for the BLD of force during upper body WAnT. However, sex did not have any effect on the BLD of force during MVICs and grip strength. The latter is in agreement with the findings of previous studies using isometric tasks. Carr et al. (22) compared maximal vs. rapidly repeated handgrip contractions, and sex did not affect the BLD of force. Ye et al. (17) explored sex differences during isokinetic elbow flexion and index finger abduction and they found no differences during elbow flexion with a −11% and −10.2% BLD of force for men and women, respectively. However, during finger abduction, males showed −13% BLD of force while females showed high variability and even a BLF of force. More recently, Kabacinski et al. (44) found no effect of sex on BLD of force during isometric knee extension.

Regarding explosive contractions, Veligekas & Bogdanis (45) studied vertical jumping in pre-pubertal boys and girls, finding that boys had 12.9% BLF while girls had a −1.6% BLD of force. The only study exploring BLD in performance during lower body cycling showed that BLD was greater in females than males, however, force values were not reported (19). The most recent study comparing the BLD of force between sexes used isokinetic repetitive leg extensions and found no significant differences between males and females. However, males showed an earlier decline in force production than females during the repetitive task (46).

Our study showed a significant influence of sex on BLD of force during the WAnTs. Males BLD increased throughout the test, from −17% at the beginning to −51% by the end. However, females only showed progressive BLD during the 3% WAnT, and during the 5% WAnT there was a BLF ranging from 6% to 11% throughout the test. Sex differences in biomechanics, anatomic, physiological, and task-related factors could also play a role in the BLD of force during the upper body WAnT. BLD of force has been related to activating type I and type II fibers. Previous research suggests that inhibition of type II muscle fibers during rapid contractions might be one potential explanation for BLD of force (1). In terms of muscle composition for males vs. females in the upper body, there is a greater distribution of type II fiber in males than in females muscles (47), which could explain why males showed a significantly greater BLD of force. Also, previous studies have shown that females have a greater resistance to fatigue during submaximal isometric contractions; however, this endurance is reduced at greater loads because males have greater muscle mass and produce greater absolute forces in the upper body (48). Also, the force-velocity relationship has been linked to the BLD, but there is no evidence of sex-related differences in these relationships (49). Antolinez et al. (26) showed that there are biomechanical differences between males and females when performing the upper body WAnT. Males held 30 degrees more neck flexion during the whole WAnT against 3, 4% and 5% of their body weight, while females increased shoulder flexion, a potential mechanism to overcome greater loads.

Some of the aforementioned factors may, in part, contribute to sex differences in the BLD of force during a WAnT, but there are no clear causes for sex differences in BLD of force. Other factors have also been shown to affect BLD, such as familiarity with the task, postural stability, limb dominance, and even neurophysiological mechanisms related to interhemispheric inhibition (17). Further research using an upper body WAnT to determine sex differences in BLD of force could help guide the development of individualized and task/participant-specific training strategies.

### Load effects on BLD

We demonstrated that BLD of force was between −13% to −16% greater at lower loads during the WAnT. However, this was only true for females. Males did not show a significant difference between the loads. To our knowledge, no previous study has explored the BLD of force using different loads. There is research that has determined the effect of load on BLD in power and performance. During bench press and countermovement jumps, some studies have suggested that load had no effect on BLD (50, 51). Ascenzi et al (52). also compared the BLD on performance during squat jumps and horizontal countermovement jumps at body weight, +25% BW and +50% BW and found that at lower loads, there was a 16% greater BLD in power. These results align with our findings, and a possible explanation for this is that unilateral performance can be highly impaired at greater loads during all-out cycling tasks. Therefore, the BLD of force phenomenon is less evident. It is important to compare these results with those of a highly trained population, specifically in upper limb-related sports, to identify if the strength and power production level will impact the BLD in force.

Previous studies exploring the effects of load on BLD have had contradictory results. Carroll et al. (53), showed that at lighter loads, the deficit is typically smaller, as the central nervous system (CNS) can more easily coordinate the simultaneous activation of both limbs. Additionally, highly trained individuals may experience a smaller BLD, even under higher loads, due to enhanced motor unit recruitment and interlimb coordination (42). Sex differences also play a role, with studies suggesting that women typically exhibit a smaller bilateral deficit compared to men, possibly due to differences in muscle fiber composition, neuromuscular activation patterns, or hormonal factors (5, 54). Overall, the load significantly affects the extent of the BLD, with heavier loads generally leading to greater deficits in force production, while sex differences, training level, and neural adaptations can further influence the magnitude of this deficit (55).

### Methodological considerations

Our study did not account for a few factors that may influence BLD, such as limb dominance, postural requirements, the set-up of the cycle ergometer, the randomization of the WAnTs and the time between WAnTs. We included mostly right hand-dominant participants, and in our results, we did not analyze if the source of the deficit was from the dominant or non-dominant limb. In terms of postural factors, even when the participant's position was standardized, capturing kinematic variables from the task would give insight into the postural modifications to complete the task and their potential effects on BLD. It is unknown as to the impact our custom-built arm-cycling ergometer has on arm cycling itself. Participants were set-up in the cycling apparatus at a comfortable distance from the crankshaft and the participant's shoulders were approximately in line with the axis of rotation of the ergometer and the elbow joint was almost in full extension (165°–175°) during the push phase. Participants' feet were strapped to the floor to minimize compensatory movements. The same position was used for each WAnT and was individualized for each participant. However, this position may not be the optimal for upper body WAnTs. Future research should determine optimal seating positions for optimal positioning during the upper body WAnT. Our experimental protocol was quasi-randomized (i.e., the participant completed either 3, 4, or 5% BW WAnT for each day but then randomly completed the unilateral and bilateral WAnTs). A true randomization may give different results. But this remains unknown. Lastly, 20 min of rest was given between the 3 WAnTs each experimental day. We cannot say with certainty that this was enough time to alleviate the development of fatigue from completing the 3 repeated WAnTs. However, Harbili (56) showed that 3 min of rest between 4 lower body 30-s WAnTs was almost enough time to mitigate fatigue (i.e., peak power remained similar but mean power had a slight decrease from WAnT 1 to WAnT 4). Thus, 20 min of rest between WAnTs should be enough recovery time to allow for optimal performance. Other measurements that could highlight neuromuscular and physiological factors of BLD during a WAnT are EMG and measures of cortical activation/inhibition. It has been suggested that a potential mechanism of interhemispheric inhibition could be related to the BLD (57). We accounted for the level of familiarity with the task, however, it will be important to include participants with different levels of fitness to identify not only the familiarity with the task but the level of activity related to the BLD of force and also in power and performance.

## Conclusions

BLD of force is present in the upper body WAnT. Sex was a factor that influenced the magnitude of this BLD in force. Males showed a similar and consistently greater BLD than females at all loads, and females showed BLF at the 5% load. The effect of fatigue on BLD also differed for males and females; males increased their deficit with fatigue, while females were highly variable without changes in BLD. Finally, we found that the BLD during an asymmetric maximal cycling contraction was more than 3 times greater than during an MVIC or a grip strength task for males. In summary, our study agrees with previous literature that establishes the BLD as being contraction-dependent. However, since this is the first study exploring the BLD of force during a maximal cycling asymmetric task, the findings merit further investigation to unveil the potential mechanisms behind the BLD during an arm cycling WAnT, which includes anatomical, physiological, metabolic, biomechanical and even psychological factors. Further investigation is warranted to determine the underlying mechanisms for sex differences of the BLD in force during a WAnT, task-specificity and the influence of fatigue.

## Data Availability

The raw data supporting the conclusions of this article will be made available by the authors, without undue reservation.
